# Longitudinal Sleep Study in Pregnancy: Cohort Profile and Prevalence and Risk Factors for Sleep Symptoms in the First Trimester

**DOI:** 10.3390/ijerph20032070

**Published:** 2023-01-23

**Authors:** Chamara V. Senaratna, Nirmala Priyadarshanie, Sharaine Fernando, Sampatha Goonewardena, Pramodya Piyumanthi, Jennifer Perret, Caroline Lodge, Garun S. Hamilton, Shyamali C. Dharmage

**Affiliations:** 1 Allergy and Lung Health Unit, Melbourne School of Population and Global Health, The University of Melbourne, Melbourne, VIC 3010, Australia; 2 Non-Communicable Diseases Research Centre, University of Sri Jayewardenepura, Nugegoda 10250, Sri Lanka; 3 Department of Community Medicine, University of Sri Jayewardenepura, Nugegoda 10250, Sri Lanka; 4 Department of Nursing & Midwifery, Faculty of Allied Health Sciences, General Sir John Kotelawala Defence University, Dehiwala-Mount Lavinia 10390, Sri Lanka; 5 Department of Physiology, University of Sri Jayewardenepura, Nugegoda 10250, Sri Lanka; 6 Monash Lung, Sleep, Allergy and Immunology, Monash Health, Melbourne, VIC 3168, Australia; 7 School of Clinical Sciences, Monash University, Melbourne, VIC 3800, Australia

**Keywords:** sleep, sleep-disordered breathing, sleep apnoea, antenatal, cohort, incidence, pregnancy

## Abstract

Sleep disorders could influence pregnancy outcomes but evidence for longitudinal associations is scarce. We established a prospective cohort of women to determine incident sleep issues and their adverse health outcomes during pregnancy and beyond, and present here the baseline cohort profile. Antenatal women in gestational weeks 8–12 were recruited (n = 535) and followed-up in each trimester and at 5–6 weeks postpartum (no attrition). Sleep symptoms and disorders were measured using STOP-Bang and Berlin questionnaires and Pittsburgh Sleep Quality Index. Incident health outcomes were extracted from clinical records. At the time of recruitment, habitual snoring was present in 13.8% of participants; “excessive sleepiness during the day” (EDS) in 42.8%; short (<7 h) sleep duration in 46.4%; “having trouble sleeping” in 15.3%; and “poor subjective sleep quality” in 8.6%. Habitual snoring was strongly associated with irregular menstrual periods for one year preceding pregnancy (*p* = 0.014) and higher BMI (*p* < 0.001). Higher age was associated with less “trouble sleeping” (OR 0.9, *p* = 0.033) and longer sleep duration was associated with better “subjective sleep quality” (OR 0.8, *p* = 0.005). Sleep issues were highly prevalent at baseline and associated with age, irregular menstruation, and obesity. This cohort will provide a robust platform to investigate incident sleep disorders during pregnancy and their effects on adverse pregnancy outcomes and long-term health of women and their offspring.

## 1. Introduction

Current maternal and child health interventions often target conventional risk factors for adverse pregnancy outcomes [1] such as micronutrient deficiencies, infectious diseases, and pregnancy-induced morbidities. However, there are other non-conventional risk factors such as sleep disorders that currently have limited or equivocal evidence but may influence adverse pregnancy outcomes. Current data comes often from cross-sectional and/or small studies [2] likely explaining the lack of interventions targeting them in antenatal health programs. Therefore, stronger evidence from longitudinal studies is urgently needed to determine the role of non-conventional risk factors to enable appropriate interventions if strong links are found between them and adverse pregnancy outcomes.

No population-based study to date has fully characterised the incidence, risk factors, and adverse pregnancy outcomes of sleep disordered breathing (SDB) or other sleep symptomatology during pregnancy in each trimester and in the postpartum period [3]. The NuMoM2b study attempted to characterise SDB but assessed this only at two time points, 6–15 weeks and 22–31 weeks of gestation [4]. Although sleep symptoms are commonly encountered in antenatal clinical practice, SDB, which is relatively common but underdiagnosed in the general population [5] and has a major bearing on the global disease burden [6], likely remains underdiagnosed in pregnancy as well. Pregnancy itself is considered a risk factor for SDB and the limited evidence available shows that the prevalence of SDB in pregnant women is variably high and increases as the pregnancy advances [7,8]. There is some evidence that the body mass index (BMI) in the first trimester and increasing maternal age [9] increases the risk for SDB in later pregnancy which could contribute to the development of pregnancy-induced hypertension, pre-eclampsia, and gestational diabetes mellitus [9,10]. Furthermore, there is scarce evidence for SDB during pregnancy influencing adverse perinatal outcomes such as intrauterine growth restriction (IUGR), preterm birth, low birthweight (LBW), and low Apgar scores [10,11]. Moreover, cohort studies investigating the association of sleep disorders and adverse pregnancy outcomes, enabling determination of temporal associations between these factors, are limited [4,8,12,13], and no population-based study has fully characterised the longitudinal links between potential risk factors for SDB, incidence of SDB during each pregnancy trimester and the postpartum period, and adverse pregnancy outcomes. Furthermore, how sleep disorders during pregnancy influence women’s health in the short or longer term has not been determined.

To address these knowledge gaps, we established a cohort of pregnant women in Sri Lanka during 2018–2020. The women were recruited at 8–12 weeks of pregnancy and followed up throughout the pregnancy and up to six weeks postpartum. By recruiting them early in the pregnancy, we aimed to determine the potential risk factors, comorbidities, and pregnancy outcomes associated with antenatal SDB and the incidence of SDB during pregnancy. We also expected to examine how SDB evolves over the antenatal to postpartum period. A longer-term objective was to explore the long-term maternal consequences of antepartum and postpartum SDB. We additionally wanted to examine how sleep symptoms (as risk markers for sleep disorders) evolve during pregnancy, as sleep symptoms constitute a significant proportion of clinical complaints during pregnancy. In this paper, we report the methods used to establish the cohort, characteristics of the cohort, and sleep symptoms and their risk factors at baseline. The incident SDB and other sleep problems throughout the pregnancy and postpartum, their comorbidities, and associated adverse pregnancy outcomes will be reported in a subsequent paper.

## 2. Materials and Methods

### 2.1. Study Area, Population and Setting

We established this population-based cohort in the capital district of Sri Lanka within four community health areas (geographical areas that have populations of 60,000–100,000 and are the grass root-level units of the national public health service delivery). This population was comprised of both rural and urban groups. Each community health area has Public Health Midwives (PHMs) who provide preventive healthcare to women and children. All pregnant women are mandatorily registered by PHMs by the 8th week of pregnancy and attend community antenatal clinics (ANCs) every month until postpartum. Each woman is issued a unique alphanumeric clinic number, which is used throughout her pregnancy for identification. We recruited our cohort from these ANCs as almost all women attend these ANCs and are easily accessible, except for a very small fraction that exclusively attend private clinics.

### 2.2. Eligibility

All women who attended the field ANCs in the selected areas, were within the first twelve weeks of pregnancy, and were aged 18 years or older were eligible. Those who had a diagnosis of hearing difficulty or visual or speaking problems, and those who had pre-existing mental illness or disability that precluded them from giving informed consent or reliable responses to the study questions were excluded.

### 2.3. Cohort Size and Sampling

We required a sample of 426 women to detect an odds ratio (OR) of 2.0 (statistical power 0.8 and two-sided alpha level of 0.05) for any pre-existing risk factors for sleep problems and for potential adverse pregnancy outcomes. We allowed for a refusal rate of 20% and consecutively recruited 535 new ANC attendees who fulfilled the eligibility criteria to obtain the required cohort size.

### 2.4. Variables and Study Instruments

#### 2.4.1. Questionnaires

We collected data on four types of variables. These were: (a) potential pre-existing risk factors for sleep, anxiety, and adverse pregnancy outcomes that included socio-demographic and economic factors, anthropometry, past medical, surgical, gynaecological and obstetric history, familial factors, and environmental exposures; (b) sleep symptoms and sleep disordered breathing risk during each trimester and postpartum; (c) common comorbidities during pregnancy such as pregnancy-induced hypertension and gestational diabetes mellitus; and (d) common adverse maternal outcomes (preterm labour, various types of assisted delivery, postpartum complications, and poor quality of life) and adverse foetal outcomes (IUGR, SGA, LBW, need for resuscitation at birth, and low Apgar score).

Data were collected using four separate composite questionnaires administered in the first, second, and third trimesters (8–12, 24–28, and 34–38 weeks of gestation, respectively) and 5–6 weeks postpartum. Each composite questionnaire consisted of multiple, validated, interviewer-administered questionnaires which focussed on different data types. Construct-specific questionnaires that were validated for the local context were used to assess sleep disorders and sleep quality, anxiety, postpartum depression, and quality of life. These were the local translations of STOP-Bang [14] and Berlin questionnaires [15], Pittsburgh Sleep Quality Index (PSQI) [16], Perinatal Anxiety Screening Scale [17], Edinburgh postnatal depression scale [18], and WHO QoL-Bref questionnaire [19]. The STOP-Bang and Berlin questionnaires and the Perinatal Anxiety Screening Scale were translated and validated as a part of the process of setting up this cohort [17,20].

#### 2.4.2. Data Extraction Forms

Four separate data extraction forms were also used at the four time points to extract data from health records. The pregnancy record issued to the antenatal women contains vital information regarding the current and previous pregnancies including clinical details, details on risk factors, management plans, referral information, and personal social and demographic information. This is updated at each contact with the health staff. This and the child health development record (CHDR) that is issued to all newborns and is similarly updated is a significant source of secondary data.

#### 2.4.3. Physical Measurements

Two physical measurements were made at the time of each interview. These were the body weight measured using calibrated electronic weighing scales and blood pressure measured using standard sphygmomanometers. Height was measured at the time of recruitment using a standard stadiometer.

#### 2.4.4. Definitions of Sleep Symptoms

Habitual snoring was defined as having loud snoring for more than 3 days per week, excessive daytime sleepiness was defined as a positive response to the question “During the past one month, did you have excessive sleepiness during the day”, and having trouble sleeping or difficulty in sleeping was defined as responding as more than three days per week to the question “How often during the past month have you had trouble sleeping” and a positive response to the question “Difficulty in sleeping even when you have the chance to sleep”. Sleep duration per day was calculated using self-reported usual time of going to bed, usual time of getting out of the bed and perceived time spent awake in the bed during the night. Perceived poor quality of sleep or dissatisfaction with sleep was derived using the questions “During the past one month, how would you rate your sleep quality overall” and “How satisfied are you with your sleep”.

#### 2.4.5. Recruitment and Data Collection Procedure at Baseline and Follow-Ups

Recruitment was started in January 2018 and the study was completed in April 2020. Recruitment was done simultaneously in all participating ANCs. All women were in their 8th to 12th week of pregnancy at the time of recruitment and baseline data collection. The second and third follow-ups were conducted during their visits to the field ANCs during 24–28 weeks and 34–38 weeks of pregnancy, respectively. The postpartum follow-ups were conducted when the women attended their field clinic 5–6 weeks postpartum.

The PHMs routinely provided information on any miscarriages, stillbirths, and neonatal deaths to the investigators. Women who underwent these events were no longer contacted due to ethical reasons. Information on such events were subsequently extracted from the records available from the PHMs. The information extracted from hospital records was also verified by cross-checking with the records available with the PHMs.

#### 2.4.6. Quality Assurance and Quality Control

We followed strict quality control measures to ensure the accuracy and reliability of data. Data collectors were nursing graduates who were trained in-house and in ANCs outside the study area. They were supervised onsite by designated supervisors whose supervisions were monitored by the local investigators. The field health staff participated in the administrative decision-making process when necessary to ensure smooth conduct of the study. A fifth (20%) of women in the cohort were randomly selected and contacted independently by the investigators over the phone within seven days of original data collection to check the reliability of interviewer-collected data. To check the reliability of data extracted from the health records, the field supervisors duplicated the data extraction for 20% of women randomly selected from the cohort within seven days of original data extraction.

Trained staff collected all biological samples and performed laboratory procedures under close supervision of a clinical physiologist. Standard quality control measures were performed for all laboratory tests.

#### 2.4.7. Ethical Approval

This study adhered to the ethical principles of the amended declaration of Helsinki. All participants provided informed written consent. Ethical approval was obtained from the Ethics Review Committee of the Faculty of Medical Sciences, University of Sri Jayewardenepura (ERC No 24/17).

#### 2.4.8. Statistical Analysis

We presented summary statistics using mean (±SD) or frequencies and percentages. The association of gynaecological, obstetric, anthropometric, and sleep related factors with common sleep issues were determined using multivariable logistic regression models. The potential confounders were mapped using a directed acyclic graph drawn based on the current knowledge and minimum sets of confounders were chosen using DAGitty [21] for inclusion in the multivariate regression models.

## 3. Results

**Figure 1:**
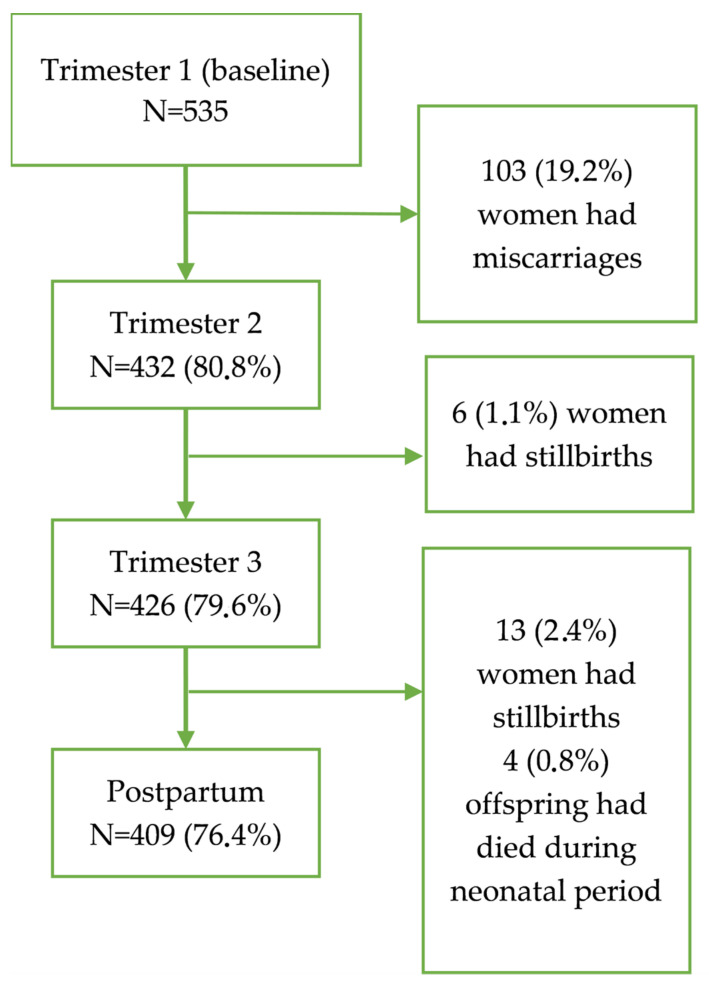
Flow chart of women who were followed up from the first trimester to postpartum and reasons for exclusion.

A total of 535 pregnant women were recruited into the study during their first trimester of pregnancy. None of the contacted women refused to participate. Figure 1 shows the follow-up of the cohort. No woman was lost to follow-up but as 103 women had miscarriages during the first trimester, only 432 women provided pregnancy-related information during the second trimester. There were six stillbirths before the next follow-up in the third trimester for which 424 women provided pregnancy-related information. The postpartum interviews were conducted for only 409 women after excluding 13 who had stillbirths and 4 whose offspring died during the neonatal period. The 126 women who lost their pregnancy or the newborn were not appreciably different from the 409 whose offspring were alive by the end of 5–6 weeks of postpartum period in terms of age at recruitment, anthropometric measurements at baseline, or the baseline prevalence of common sleep symptoms (*p* > 0.05 for all; data not shown). All 535 women and 409 live offspring were available for further follow-ups after the postpartum period.

Table 1 shows the basic sociodemographic characteristics of the cohort at the baseline. Their mean (±SD) age was 29.8 (±5.09) years with 20.7% being aged 35 years or older. Less than one fifth had bachelor’s or higher degrees. Their median income was LKR 60,000 (range LKR 5000–550,000; cf. national median of LKR 43,511 [22]).

About half of the cohort were primigravida (Table 1). Most (63.9%) of the multigravida had one living child. Only 29.0% had ever used a contraception method. Over 80.0% reported having regular menstrual cycles within the year prior to pregnancy.

They had a mean BMI of 22.8 kg/m^2^ at the time of recruitment; 12.5% (*n* = 67) were underweight (<18.5 kg/m^2^), 24.5% (*n* = 131) were overweight (25.0 kg/m^2^ < BMI < 30 kg/m^2^), and 3.9% (*n* = 21) were obese (BMI > 30 kg/m^2^). The mean neck circumference was 34.4 cm. Changes in the mean BMI and neck circumference across the follow-up period are shown in Table 2. Both measures increased until the 3rd trimester and then decreased during the postpartum period. Some women had existing chronic diseases at baseline; nineteen (3.5%) had asthma, fourteen (2.6%) had thyroid disease, twelve (2.2%) had diabetes mellitus; five (0.9%) had hypertension, three each had cardiac disease (0.6%) and renal disease (0.6%), and twelve (2.2%) had other miscellaneous conditions. The mean score (±SD) for the perinatal anxiety screening scale was 14.9 (±9.5) with 120 women (14.5%) having moderate to severe anxiety level.

Of the 409 women whose data were available across all follow-ups, 15.4% had gestational diabetes mellitus and 10.0% had gestational hypertension (Table 3). Over half of the 409 women had their babies delivered by caesarean section and nearly 10.0% had prolonged labour. Nearly 14.0% of deliveries were preterm and 17.0% had low birth weight.

### 3.1. Prevalence of Common Sleep Issues at Baseline (First Trimester)

Seventy-four women (13.8%) reported habitual snoring at baseline and 42.8% (*n* = 229) reported excessive daytime sleepiness. The mean (±SD) sleep duration was 6.8 (±5.06) h. Close to half (46.4%, *n* = 248) slept for less than seven hours on average, 15.3% (*n* = 82) reported having trouble sleeping despite having enough opportunities to sleep, and 8.6% (*n* = 46) indicated poor quality sleep or unhappiness about their sleep. Sixty-seven women (12.5%) had STOP-Bang scores of three or higher, and sixteen women (3.0%) had two or more positive categories in the Berlin questionnaire.

### 3.2. Association of Common Sleep Issues with Gynaecological, Obstetric, Anthropometric, and Sleep Related Factors

Table 4 shows how selected risk factors were associated with sleep issues at the baseline survey. Irregular menstrual periods for one year preceding the pregnancy and higher BMI were strongly associated with habitual snoring (*p* = 0.014 and *p* < 0.001, respectively). Having trouble sleeping, duration of sleep, or quality of or satisfaction with sleep was not associated with EDS (*p* > 0.05 for all). Higher age and moderate or severe anxiety at the baseline survey were associated with trouble sleeping (OR 0.9, *p* = 0.033 and OR 3.9, *p* < 0.001, respectively) but having trouble sleeping was not associated with sleep duration (*p* > 0.05). Longer sleep duration showed a protective effect (OR 0.8, *p* = 0.005) on poor subjective sleep quality or dissatisfaction with sleep.

## 4. Discussion

There is paucity of prospective studies on the association of SDB with adverse pregnancy outcomes, which can have significant policy implications. Therefore, we established this cohort to investigate the prevalence and incidence of common sleep issues and the incidence of SDB in pregnancy, their risk factors, and the maternal and offspring outcomes. Despite the NuMoM2b study [4] assessing SDB at two time points during pregnancy, ours is the first cohort study that aimed to investigate the incidence of and a wide range of risk factors for SDB in pregnancy and their adverse outcomes. We based this cohort in the general population to ensure that our findings are generalisable. In this cohort profile paper, we have reported the gynaecological and obstetric profiles, sleep symptoms, and their risk factors at the time of recruitment in the first trimester. We found that a high proportion of women were having common sleep problems as early as in the first trimester of pregnancy. Age, BMI, irregular menstrual cycles during one-year preceding pregnancy, and insufficient sleep duration were risk factors for some of these sleep issues.

As future investigations using data from this cohort will closely examine the association between reproductive health practices/outcomes and sleep, reporting the profile of this cohort in this regard is important. Seven in ten women in this cohort had never used contraception and are not dissimilar to the women in low- and lower-middle-income countries in this regard [23]. The fact that nearly one fifth of the total cohort have used some type of hormonal contraception prior to their pregnancy is important as the changes in the level of reproductive hormones in the body is associated with sleep issues [24]. Although the stillbirth rate in previous pregnancies experienced by the multigravida in this cohort was nearly double the national figure of 0.6%, it was less than the average stillbirth rates reported for the world in general (13.9%) and the south Asian region (18.2%) [25] possibly reflecting the better maternal care services that were available to the women in this cohort. However, the stillbirth rate in the total cohort during the follow-up period far exceeded the multigravida’s previous rate as well as the national rate and is a concern, the causes of which remain to be investigated in future analyses.

Both underweight and overweight/obesity are potential risk factors for adverse pregnancy outcomes [26]. The proportion of underweight women in this cohort was slightly higher than the median underweight for the low- and low-middle-income countries in 2005 (5.9–9.3%) [27] but lower than the 15.5–22.5% reported for south and southeast Asian countries in 2019 [28], possibly a reflection of better health and welfare services available to them. Nevertheless, this is still likely to have significant health implications for pregnancy outcomes in this cohort [26]. Over 28% were overweight or obese and corresponds to the gradually increasing pre-pregnancy overweight and obesity trends in low- and lower-middle-income countries [28]. The risk of SDB increases with increasing BMI and so do other sleep issues such as habitual snoring and difficulty in sleeping [10]. It also increases the risk of respiratory conditions such as asthma [29] as well as the risk of gestational diabetes mellitus and hypertension [30]. However, the proportion of gestational diabetes mellitus and hypertension reported in our cohort during the follow-up period compares well with the average global prevalence of these conditions, i.e., 14.7% [31] and 4.1–19.4% [32], respectively. The mean BMI gradually increased over the duration of pregnancy and decreased after delivery as expected and remained higher at 6 weeks post-partum than that in the first and second trimesters of pregnancy. Neck circumference followed a similar trend. Both these potentially increased the risk of habitual snoring and SDB post-pregnancy as well as other cardiometabolic diseases for which high BMI is a risk factor [33].

Some delivery-related outcomes in this cohort are also of interest. Over half of the women in this cohort had caesarean sections which was much higher than the estimated global average of 21%, 27% for the developed world and 8–24% for the developing world [34] and was in clear excess of what is considered as a beneficial proportion (19%) [35]. This is a concern given that any selection bias in our cohort is unlikely. Most women who attend these ANCs deliver in state hospitals where caesarean sections are performed only when clinically indicated; the number of women (<5%) who deliver in private hospitals and can opt to have a caesarean section without a clinical indication is small and cannot significantly contribute to this high proportion. Therefore, this needs to be investigated in future analyses. The proportion of preterm deliveries in our cohort was higher but not very different from the estimated global prevalence of 11% [36]. Despite maternal underweight being relatively lower in our cohort than that in the region, the proportion of low birth weight was similar to that in other developing countries (median 15.9%, range, 9.0–35.1%) [37].

Sleep complaints in pregnancy are common in clinical practice. However, habitual snoring in our cohort at the baseline was much higher than the 7% reported in Sweden [38] and Saudi Arabia [39], and 9% [13] reported in the USA. The questions and criteria used in the latter two studies [13,39] to determine snoring were similar to what we used and are unlikely to account for this difference. A part of this high proportion in our cohort is likely due to overweight and obesity as shown by the strong association that was present between habitual snoring and higher BMI (*p* < 0.001) and confirms previous evidence for an association between higher BMI and habitual snoring in pregnancy [38]. However, given that the prevalence of obesity in the three cited studies were not lower than that in our cohort, overweight and obesity alone does not explain this higher prevalence of snoring in our cohort. Temperature and relative humidity that have different effects on snoring and SDB [40] could have played a role but we do not have data to confirm this. Having irregular menstrual periods for one year preceding pregnancy was also associated with habitual snoring (*p* = 0.020). This association was independent of overweight and obesity. Oestradiol influences the development of muscles in the upper respiratory tract [41] and fat distribution [42], and low female sex hormone levels (such as post-menopause) are associated with an increased risk for SDB. It is possible that irregular periods are indicative of lower oestrogen levels in this cohort, which could explain the association with habitual snoring; however, this is speculative and cannot be confirmed as we were not able to measure pre-pregnancy sex hormone levels. Advanced age is also a reported risk factor for snoring in our cohort. A surprisingly high proportion reported EDS in our cohort, which is much higher than the 31–33% that has been previously reported in other studies in Sweden [43] and USA [44]. Both snoring and EDS suggest a high probability of SDB [6] and the higher proportions of both in our cohort suggest the likelihood of high proportion of SDB in this cohort. However, we have not fully characterised SDB as measured by STOP-Bang and Berlin questionnaires except for reporting their baseline prevalence in this paper as this will be a part of a separate paper that will investigate the incidence, evolution, and risk factors for SDB during pregnancy. As expected, EDS at baseline was associated with having trouble sleeping (OR 1.6; 95% CI 1.01, 2.6).

The mean (±SD) sleep duration of 6.8 (±5.06) h in this cohort reflected unsatisfactory sleep time [44] overall. Nearly half of the cohort reported sleep durations of less than seven hours which was much higher than what was previously reported (26%) [44]. Recruitment for that study, however, was institution- and convenience-based and selective whereas ours is a population-based cohort, which could explain the observed difference in the proportions. Short sleep duration is an important risk factor for chronic diseases including diabetes mellitus in pregnant women [45] and thus increases this risk for our cohort. Interestingly, higher age protected the women from having trouble sleeping. This protective effect was small but significant and it is unclear if any socio-cultural elements influenced this association, given that previous studies on sleep quality in pregnant women have indicated increased age was a risk factor for poor sleep quality [46,47]. On the other hand, having trouble sleeping is only one dimension of sleep quality and several other dimensions such as sleep latency, insomnia, and sleep duration were shown to lack an association with age depending on the trimester of pregnancy in which they were assessed [48]. Having trouble sleeping was not associated with duration of sleep (*p* = 0.207). However, longer duration of sleep protected women from having poor subjective sleep quality or dissatisfaction with sleep (*p* = 0.005); the latter was reported by fewer than one in ten women (cf. nearly 40–45% previously reported) [44,49] despite higher proportions of other sleep symptoms. This lower proportion of dissatisfaction with sleep (while sleep symptoms remain highly prevalent) likely represents the low priority given to sleep issues in the local context.

This cohort was recruited early in their pregnancy, as close as possible to the pre-pregnancy period, as recruiting them during pre-pregnancy period and following up was logistically and financially not viable. Many physical and sleep-related changes that occurred during pregnancy such as weight gain and difficulty in sleeping are not very apparent during the first trimester. This early recruitment allowed us to determine the evolution of health conditions and symptoms over the entire period of pregnancy and after delivery. To ensure the validity of the data, we translated and validated internationally used and cross-culturally validated standard questionnaires as a part of setting up this cohort. These findings are published elsewhere and confirmed the validity of the STOP-Bang and Berlin questionnaires in detecting SDB in pregnancy [20] and of perinatal anxiety screening scale in detecting anxiety in pregnancy [17] in the local context. The data collected on pre-existing potential risk factors allowed us to determine their longitudinal associations. However, the cohort size that was calculated as per the main outcomes may not have adequate statistical power to detect other rare associations. Lack of sleep study data for SDB and clinical diagnoses for anxiety is also a limitation, but this is alleviated to some extent by the use of validated questionnaires. The quality assurance process and zero loss to follow-up will ensure that the cohort provides valid and reliable data. The involvement of health staff in some activities and in field-level decision-making ensured their cooperation and facilitates future follow-up of the cohort in the field. Finally, the last month of the follow-up period coincided with the beginning of the COVID-19 pandemic-related service and social disruptions. Although this may have affected some information collected in the study such as anxiety measures, given that the data collection was conducted over a period of 27 months and only a few women were followed up during this last month, it is unlikely that any COVID-19 related issues materially affected our findings.

## 5. Conclusions

Baseline sleep symptoms were highly prevalent in this cohort of pregnant women suggesting that this cohort is at high risk for SBD. Given the likely under-reporting of underlying sleep disorders that potentially influence pregnancy outcomes and comorbidities and long-term health of women, we recommend that physicians who provide routine care actively look for and explore sleep symptoms in antenatal women. Older age, higher BMI, and a history of irregular menstrual cycles immediately preceding pregnancy could be used to flag particularly susceptible women during antenatal visits for further action where appropriate. The data from this cohort will allow us to determine the incidence of sleep disorders and anxiety during pregnancy, and their association with the incidence of adverse pregnancy outcomes and longer-term health outcomes in later life. The offspring of this cohort of women now have comprehensive profiling of their antenatal period and will be a vital parallel cohort to investigate their longer-term health outcomes.

## Figures and Tables

**Table 1:** Basic socio-demographic and reproductive health characteristics of the cohort at baseline (*n* = 535).

	Characteristic	*n*	%
Age (years)			
	<35	424	79.3
	≥35	111	20.7
School education			
	8 years or less	33	6.3
	9–11 years	180	33.7
	12–13 years	322	60.2
Tertiary education/Professional qualifications			
	None	242	45.2
	Certificates or diplomas	198	37.0
	Bachelor’s degree or above	95	17.8
Current occupation			
	Professional, technical, administrative, or managerial	128	23.9
	Clerical or related	87	16.3
	Agriculture, fisheries, or other elementary	19	3.6
	Unemployed or housewives	301	56.3
Average monthly household income (LKR) ^1^			
	0–50,000	221	41.4
	50,001–100,000	225	42.0
	>100,000	89	16.6
Gravidity			
	Primigravida	272	50.8
	Multigravida	263	49.2
Number of living children (*n* = 263)			
	One child	168	63.9
	Two children	60	22.8
	Three or more children	35	13.3
H/O miscarriages (*n* = 263)		91	34.6
H/O stillbirths (*n* = 263)		3	1.1
Ever used contraception		155	29.0
Regular menstrual cycles during the preceding one year		431	80.6

^1^ Median household income in Sri Lanka is LKR 43,511 [22].

**Table 2:** Change in the body mass index (BMI) and neck circumference in women from the first trimester to postpartum period.

	Trimester 1 (8–12 Weeks)	Trimester 2 (24–28 Weeks)	Trimester 3 (34–38 Weeks)	Postpartum (5–6 Weeks)
	Mean ± SD	Mean ± SD	Mean ± SD	Mean ± SD
BMI (kgm^−2^)	22.8 ± 3.7	23.9 ± 3.7	25.6 ±3.5	24.2 ±3.3
Neck circumference (cm)	34.4 ± 3.2	34.9 ± 3.2	35.5 ± 3.1	35.0 ± 3.1

**Table 3:** Key pregnancy outcomes for women who completed all follow-ups (*n* = 409).

	Outcome	*n*	%
Pregnancy-specific comorbidities			
	Pregnancy-induced hypertension	41	10.0
	Gestational diabetes mellitus	63	15.4
Mode of delivery			
	Normal vaginal delivery	193	47.2
	Assisted delivery (Forceps/vacuum)	9	2.2
	Caesarean section	207	50.6
Prolonged labour		40	9.8
Premature rupture of membranes		18	4.4
Gestational age at birth			
	Preterm (<37 weeks)	57	13.9
	Normal (37–40 weeks)	345	84.4
	Post-term (>40 weeks)	7	1.7
Foetal outcomes			
	Intrauterine growth restriction	6	1.5
	Low birth weight (Birth weight < 2.5 Kg)	71	17.4
	Neonatal intensive care unit admission	16	3.9
	Breastfeeding not established within 30 min of birth	36	8.8
Postpartum outcomes			
	Postpartum haemorrhage	19	4.7
	Breastfeeding problems	44	10.8
	Postpartum Infections	4	1.0
	Deep-vein thrombosis	2	0.5

**Table 4:** Risk factors for common sleep symptoms during first trimester.

Sleep Symptom during First Trimester	Risk Factor	Unadjusted OR (95% CI)	*p*	Adjusted OR (95% CI)	*p*
Habitual snoring					
	Age at menarche ^1^	1.00 (0.8, 1.2)	0.996	-	-
	Duration of hormonal contraceptive use ^1^	1.00 (1.0, 1.0)	0.131	-	-
	Irregular menstrual periods for one year preceding pregnancy ^2^	2.1 (1.2, 3.7)	0.015	2.0 (1.1, 3.8)	0.020
	Gravidity ^3^	0.9 (0.7, 1.2)	0.588	0.9 (0.6, 1.2)	0.308
	Neck circumference ^4^	1.2 (1.1, 1.3)	<0.001	1.1 (1.0, 1.2)	0.271
	Body mass index (kg/m^2^) ^5^	1.2 (1.1, 1.2)	<0.001	1.2 (1.1, 1.2)	<0.001
	Age (years)	1.0 (1.0, 1.0)	0.122	-	-
	Previous surgeries of nose, mouth, throat, or neck ^1^	1.3 (0.4, 4.8)	0.644	-	-
Excessive daytime sleepiness					
	Having trouble sleeping ^6^	1.2 (1.04, 2.7)	0.032	1.6 (1.01, 2.7)	0.042
	Sleep duration (hours) ^7^	1.0 (1.0, 1.0)	0.651	1.0 (1.0, 1.0)	0.453
	Subjective sleep quality or satisfaction with sleep ^8^	0.7 (0.4, 1.3)	0.304	0.9 (0.5, 1.8)	0.812
Having trouble sleeping					
	Age (years) ^1^	0.9 (0.9, 0.9)	0.033	-	-
	Body mass index (kg/m^2^) ^5^	0.9 (0.9, 1.0)	0.116	1.0 (0.9, 1.0)	0.203
	Gravidity ^9^	0.9 (0.7, 1.2)	0.515	1.0 (0.8, 1.3)	0.989
	Moderate or severe anxiety ^10^	3.9 (2.3, 6.4)	<0.001	3.9 (2.4, 6.4)	<0.001
Duration of sleep (hours)					
	Age (years) ^1^	−0.03 (−0.1, 0.1)	0.503	-	-
	Body mass index (kg/m^2^) ^5^	0.1 (−0.05, 0.18)	0.286	-	-
	Gravidity ^3^	−0.4 (−0.9, 0.01)	0.048	−0.06 (−0.05, 0.18)	0.298
	Leg twitch/jerk in sleep ^11^	−0.4 (−2.5, 1.7)	0.731	−0.3 (−2.4, 1.8)	0.760
	Having trouble sleeping ^6^	−0.8 (−2.0, 0.4)	0.181	−0.8 (−2.0, 0.4)	0.187
Poor subjective sleep quality or dissatisfaction with sleep					
	Age (years) ^1^	1.0 (1.0, 1.1)	0.187	-	-
	Body mass index (kg/m^2^) ^5^	1.0 (0.9, 1.0)	0.444	-	-
	Gravidity ^3^	1.1 (0.9, 1.5)	0.351	1.3 (0.9, 1.8)	0.127
	Duration of sleep (hours) ^12^	0.7 (0.6, 0.9)	<0.001	0.8 (0.7, 0.9)	0.005

^1^ No adjustments required; ^2^ adjusted for body mass index, age at menarche, and duration of hormonal contraceptive use; ^3^ adjusted for age, duration of hormonal contraceptive use; ^4^ adjusted for body mass index, and duration of physical exercise; ^5^ adjusted for age at menarche, duration of hormonal contraceptive use, irregular menstrual periods for one year preceding pregnancy, and duration of physical exercise; ^6^ adjusted for age, body mass index, gravidity, nasal allergies, and duration of physical exercise; ^7^ adjusted for age, body mass index, age at menarche, duration of hormonal contraceptive use, irregular menstrual periods for one year preceding pregnancy, gravidity, leg twitch/jerk during sleep, having trouble sleeping or difficulty in sleeping, nasal allergies, and duration of physical exercise; ^8^ adjusted for age, body mass index, neck circumference, age at menarche, duration of hormonal contraceptive use, irregular menstrual periods for one year preceding pregnancy, gravidity, leg twitch/jerk during sleep, having trouble sleeping or difficulty in sleeping, nasal allergies, duration of sleep, and duration of physical exercise; ^9^ adjusted for age and duration of hormonal contraceptive use; ^10^ adjusted for age and gravidity; ^11^ adjusted for age, body mass index, neck circumference, age at menarche, duration of hormonal contraceptive use, irregular menstrual periods for one year preceding pregnancy, and gravidity; ^12^ adjusted for age, body mass index, neck circumference, age at menarche, duration of hormonal contraceptive use, irregular menstrual periods for one year preceding pregnancy, gravidity, leg twitch/jerk during sleep, having trouble sleeping or difficulty in sleeping, nasal allergies, and duration of physical exercise.

## Data Availability

All manuscript-related data is available in the Tables and Figure. Further information can be obtained from the corresponding author.
